# Microbicidal mechanisms for light-activated molecular nanomachines in *Mycobacterium smegmatis*: A model for pathogenic bacteria

**DOI:** 10.1016/j.onano.2025.100240

**Published:** 2025-02-25

**Authors:** Thushara Galbadage, Dongdong Liu, James M. Tour, Jeffrey D. Cirillo, Richard S. Gunasekera

**Affiliations:** a Department of Microbial Pathogenesis and Immunology, Texas A&M University, School of Medicine, Bryan, TX, USA; b Department of Chemistry Rice University, Houston, TX, USA; c Department of Materials Science and NanoEngineering, Rice University, Houston, TX, USA; d Smalley-Curl Institute, Rice University, Houston, TX, USA; e NanoCarbon Center, Rice University, Houston, TX, USA; f Department of Chemistry, Rice University, Houston, TX, USA; g Center for Airborne Pathogens Research and Imaging, Texas A&M Institute for Genome Sciences and Society, Bryan, TX, USA; h Department of Chemistry and Biochemistry, Biola University, La Mirada, CA, USA

**Keywords:** Nanomolecules, Molecular machines, Multidrug-resistant, Mycobacteria, *M. tuberculosis*, *M. smegmatis*, *S. aureus*, *E. coli*

## Abstract

There is a global health crisis of antimicrobial resistance, responsible for over a million deaths annually. Mycobacterial infections are a major contributor to this crisis, causing more deaths than any other single infectious agent. Notably, the rise of multidrug-resistant (MDR), extensively drug-resistant (XDR), and totally drug-resistant (TDR) strains of *Mycobacterium tuberculosis* has led to higher mortality rates and challenge all existing antibiotic regimens. Light-activated molecular nanomachines (MNMs) represent a promising class of broad-spectrum antimicrobial agents that could help counter this rise in antimicrobial resistance. Addressing a key knowledge gap, this study explores the mechanisms of action for MNMs in *Mycobacterium smegmatis*, a surrogate model for pathogenic mycobacteria. We show that fast-rotor MNMs significantly reduce bacterial viability, achieving up to 97 % reduction in *M. smegmatis* with 30 minutes of light activation when compared to non-activated MNM **1** (*p* < 0.0001, *t* = 24.55), as determined by an unpaired *t*-test. Using fluorescence and confocal microscopy, we also show the colocalization of MNM **1** with *M. smegmatis* as part of their mechanism of action. The ability to translate these observations to pathogenic mycobacteria was demonstrated by the ability of MNM **1** to kill 93.5 % of *M. tuberculosis* with 5 minutes of light activation when compared to non-activated MNM **1** (*p* < 0.0001, *t* = 19.24). These findings suggest that MNMs have the potential to be innovative and sustainable antimicrobial agents for the treatment of pathogenic mycobacterial infections.

## 1. Introduction

Antimicrobial resistance is a growing global health crisis with profound medical and public health implications [1,2]. The persistence and rise of antimicrobial resistance pose a significant public health threat as evidenced by over a million deaths annually attributed to antimicrobial-resistant (AMR) pathogens [1]. If current rates of antimicrobial resistance continue, it is projected that over 10 million annual deaths will be caused by AMR pathogens by 2050 [3].

Mycobacterial infections are one such challenging global threat with several species of mycobacterial pathogens emerging as major concerns, particularly due to the rise in antibiotic resistance and multidrug-resistant strains [4–6]. *Mycobacterium tuberculosis*, the causative agent of tuberculosis, and *Mycobacterium leprae*, responsible for leprosy, are important pathogens causing chronic and debilitating infections in humans. The ability of these bacteria to develop resistance to multiple drugs poses a serious obstacle to currently effective treatments, necessitating prolonged and often complex therapeutic regimens [7]. Multidrug-resistant (MDR), extensively drug-resistant (XDR), and totally drug-resistant (TDR) strains of *Mycobacterium tuberculosis* are some of the most important public health challenges worldwide, and perhaps even more important than ESKAPE pathogens [7–9]. MDR, XDR, and TDR lead to higher mortality rates and challenge all existing antibiotic regimens [7,10]. The rapid development of MDR, XDR, and TDR strains further exacerbates the difficulty in managing mycobacterial infections, making it imperative to address antibiotic resistance comprehensively.

There are three main mechanisms that pathogens use to develop antimicrobial resistance. First, the accumulation of various resistance genes or pathogenicity islands through mobilization and horizontal gene transfer from various environmental bacteria. Second, random mutants that occur in antibiotic target genes that make them have fewer active sites for antibiotic action. Third, upregulation of inherent resistance mechanisms, including antibiotic inactivating enzymes and efflux system to pump out antibiotics that enter the pathogen [11,12]. These antimicrobial resistance mechanisms can render all conventional and targeted antimicrobial agents ineffective over time. Therefore, innovative antimicrobial agents that can circumvent antimicrobial resistance mechanisms must be developed.

**Fig. 1.**
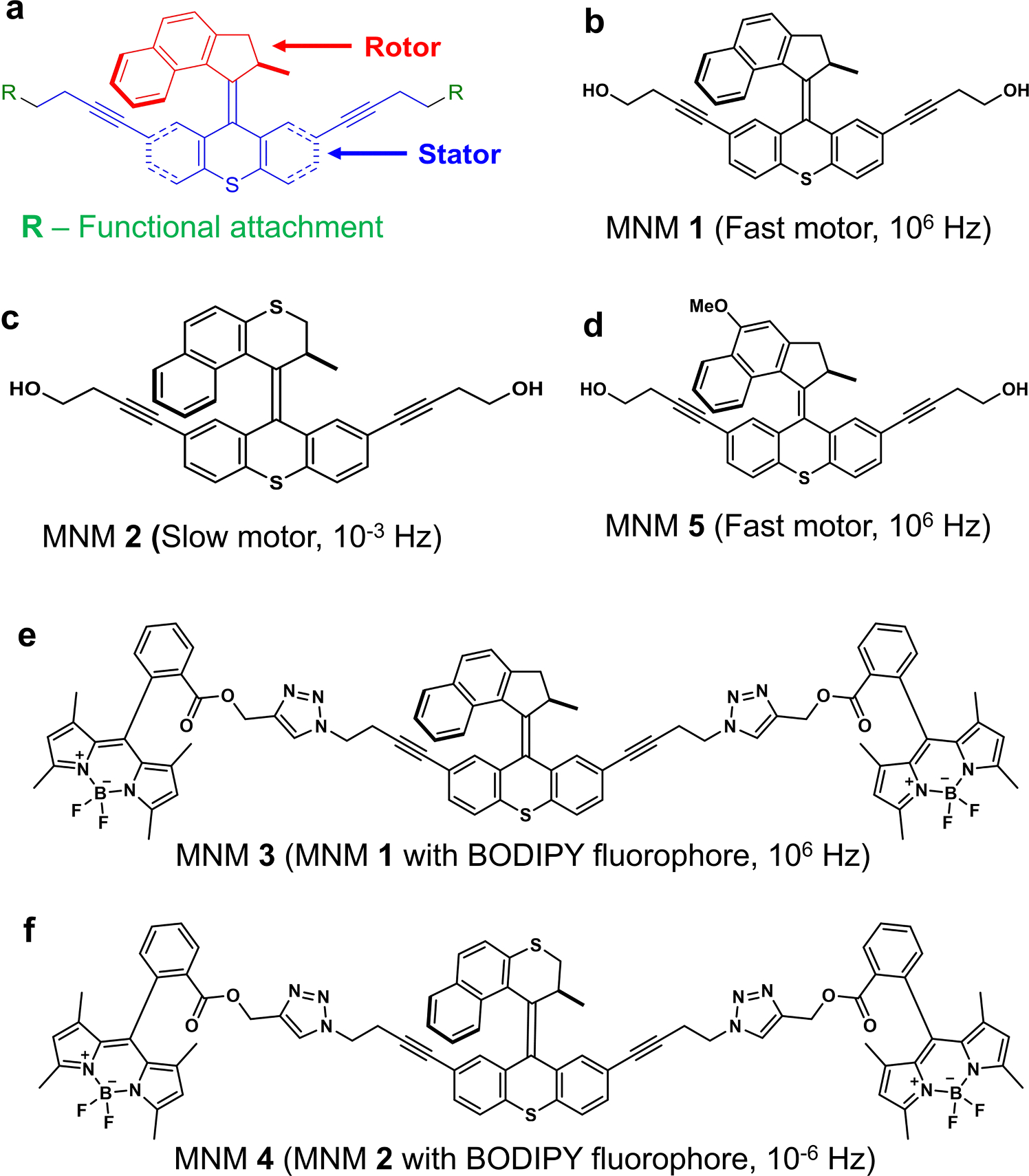
Molecular nanomachines (MNMs) were used in this study. (a) A graphic representation of the MNM structure showing the rotor and stator components. (b) MNM **1** is a fast motor with a rotor that can rotate at 2–3 MHz when activated with 365 nm light. (c) MNM **2** is a slow motor with a rotor that can rotate a 1.8 revolutions per hour when activated with 365 nm light. (d) MNM **5** is a fast motor with a rotor that can rotate at 2–3 MHz when activated with 395 nm light. (e) MNM **3** fast Motor with BODIPY fluorophore attached to 2 arms in its stationary component and activated by 365 nm light. (f) MNM **4** slow motor with BODIPY fluorophore attached to 2 arms in its stationary component and activated by 365 nm light.

To this end, our collaborative research group has characterized molecular nanomachines (MNMs) as a unique class of nanomechanical broad-spectrum antimicrobial agents that have the potential to remain effective long-term [13,14]. MNMs are synthetic organic nanomolecules that can rotate unidirectionally up to several million rotations per second upon light-activation (Fig. 1) [15–18]. Our approach using MNMs bypasses the need for specific pathogen interactions and likely renders the pathogen incapable of acquiring resistance to these MNM agents. More recently, MNMs were shown to effectively eliminate antibiotic-resistant Gram--negative and Gram-positive bacteria, including methicillin-resistant *Staphylococcus aureus*, while preventing resistance development in an *in vivo* model of burn wound infection [14].

In recent studies, the mechanism of action of MNMs has been explored, focusing on the physical membrane disruption and damage done to bacterial or eukaryotic cells. Electron microscopy (EM), membrane permeability assays, and RNA-seq methods were used to characterize the effects of MNMs on bacterial cells [13,14]. While these methods illustrated various aspects of MNM activity, the mechanism of action of these MNMs is not yet fully understood, posing an important knowledge gap that needs to be addressed. Here we use *Mycobacterium smegmatis* as a model for mycobacterial species to show that light-activated MNMs can act as antibacterial agents against mycobacteria, reduce bacterial viability, and further characterize the mechanism of action of MNMs. *Mycobacterium tuberculosis, Staphylococcus aureus*, and *E. coli* were used as relevant pathogenic mycobacterial species, a Gram-positive control and a Gram-negative control, respectively.

*M. smegmatis* is an acid-fast staining bacterium with a lipid-rich cell wall containing mycolic acid [19]. While *M. smegmatis* are non-pathogenic free-living mycobacteria, there are several related pathogenic mycobacteria, such as *M. tuberculosis, M. avium,* and *M. leprae* that are of significant clinical importance [20]. Tuberculosis caused by *M. tuberculosis* is the leading cause of death worldwide due to a single infectious agent and the ninth leading cause of death overall [21]. *M. tuberculosis* has MDR and XDR strains that have significant implications in immune-compromised patient populations [22]. *M. smegmatis* is a relevant and commonly used mycobacterium model to study cellular processes that are related to the *M. tuberculosis* pathogen [20].

In this study, we exposed *M. smegmatis* to light-activated MNMs, observed the reduction in bacterial viability, and characterized their nanomechanical action using fluorescently labeled MNMs, and confocal microscopy. Here we show that fast motor MNMs can reduce mycobacterial viability, with their nanomechanical action by colocalizing with the bacteria. Our study seeks to further explore the use of MNMs to counter antibiotic resistance, safeguard public health, and develop innovative interventions for a sustainable and resilient future in the face of the formidable global health threat of antibiotic resistance.

## 2. Methods

### 2.1. Bacterial strains

A wild-type *Mycobacterium smegmatis* (mc^2^155), a mutant *Mycobacterium tuberculosis* (mc^2^7000), a bioluminescent strain of *Staphylococcus aureus* (Xen36) (ATCC 49525:: luxABCDE operon), and wild-type *E. coli* strains (K-12, HB101) were used to evaluate the bactericidal properties of MNMs against these bacterial species. A tdTomato fluorescent *M. smegmatis* strain (ψms23) was derived by transforming a wild-type *M. smegmatis* (mc^2^155) with a multi-copy plasmid, pJDC60 (pFJS8ΔGFP::tdTomato, under a PL5 promoter, with kanamycin selection). The fluorescent strain of *M. smegmatis* was used for confocal microscope imaging to explore the colocalization of MNMs and bacteria.

### 2.2. Molecular nanomachines

MNMs are molecular motors that have a stator and a rotor component, with different functional groups attached to the stator (Fig. 1a). MNM **1** is a fast motor that rotates at about 2–3 × 10^6^ revolutions per second (10^6^ Hz) (Fig. 1b). MNM **2** is a slow motor that rotates at about 1.8 revolutions per hour (10^−3^ Hz) (Fig. 1c). MNM **2** served as a non-rotating analog to MNM **1**. MNM **3** and MNM **4** are MNM **1** and MNM **2,** respectively, with two BODIPY fluorophores attached to the stator (Fig. 1e and f). MNMs **3** and **4** were used for confocal microscope imaging. MNMs **1** to **4** are activated with 365 nm light. MNM **5** is a fast motor that rotates at about 2–3 × 10^6^ revolutions per second (10^6^ Hz) and activated with 395 nm light (Fig. 1d, Supplemental Figure 1).

### 2.3. Bacterial viability assays with 365 nm light-activated MNMs

**Fig. 2.**
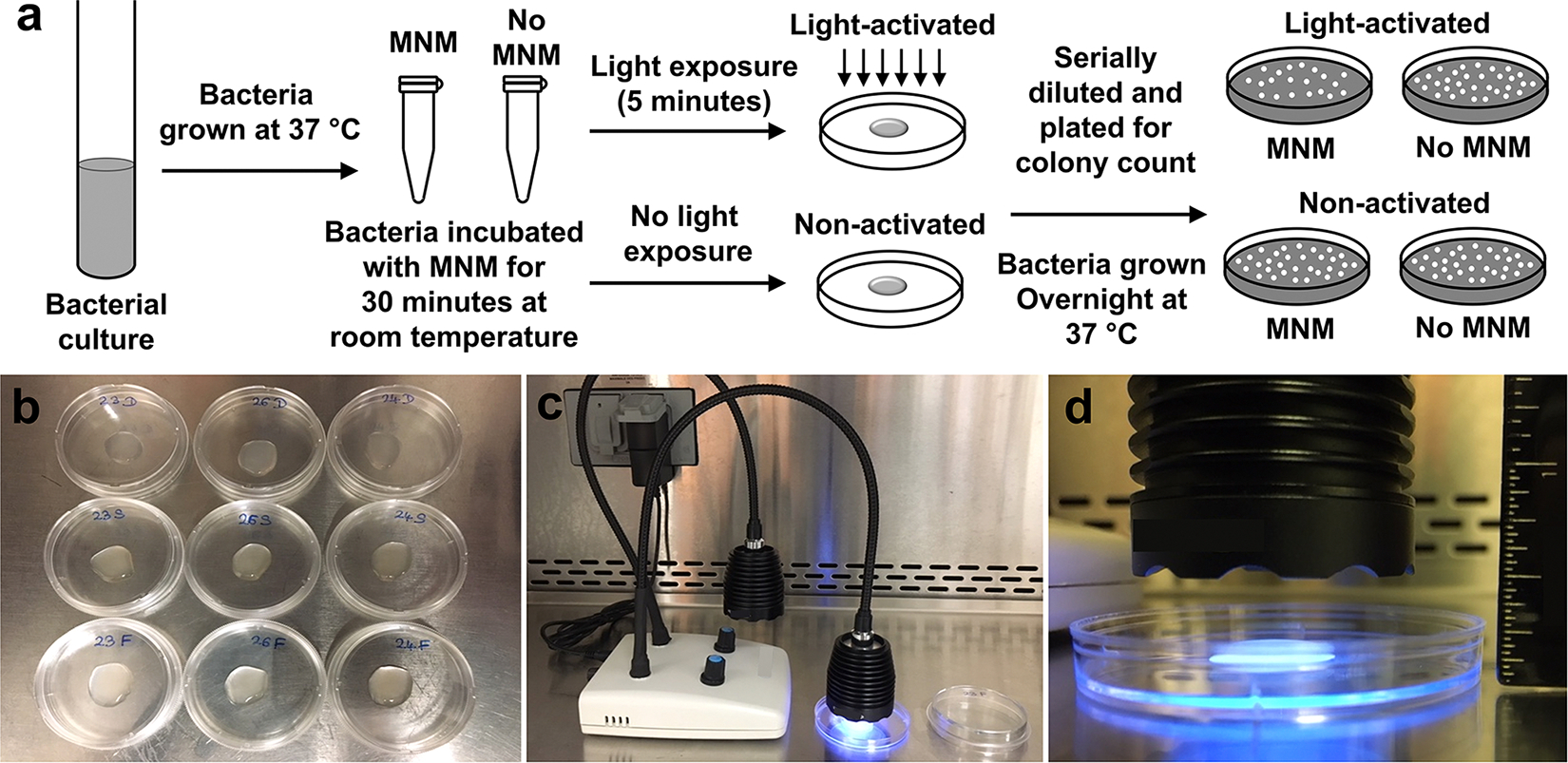
Bacterial viability assay with light-activated molecular nanomachines (MNMs) Setup. (a) Experimental setup for bacterial viability reduction assays. A log growth phase bacterial culture was incubated with no MNM (with dimethyl sulfoxide, DMSO), MNM **2**, or MNM **1** for 30 minuntes, activated with 365 nm light for 5 minutes, and plated for CFU/mL counts. (b) 120 μl of bacterial culture was placed on the lid of a petri dish for light activation. (c) 365 nm light source used to activate MNMs **1** to **4**. Bacterial cultures were exposed to 365 nm light for 5 minutes. (d) The light source was placed directly above the bacterial culture at a constant distance from the culture (1.3 cm or 0.5 inches), to ensure uniform and constant energy delivery.

Overnight cultures of bacteria grown in either Luria-Bertani (LB) or Middlebrook with ADC (MADC) media were used to start secondary cultures. Cultures were placed in a shaker at 37 °C for about 4 h to obtain fresh bacterial cultures in the log phase (Fig. 2a). Serial dilutions of cultures were made using 100 μL of culture with 900 μL of 1x PBS. Two 1 ml dilutions of 10^−4^ or 10^−5^ cultures were used for the viability assays. The concentration of MNMs in 1 % DMSO was either 1 or 10 μM. A 1 μM concentration of MNMs was used for *M. smegmatis, M. tuberculosis*, and *S. aureus*. 10 μM concentration of MNMs was used for *E. coli*. Using 10 mM working stock of MNM, 1 μL was added to 1 ml of bacterial culture at 10^−5^ to give a 10 μM concentration. 1 μL of DMSO without MNM was added to the second 1 ml bacterial culture at 10^−5^ as the control (No MNM control). They were incubated for 30 minutes on a test tube rocker at room temperature. At 30 minutes after incubation, 120 μl of the culture of each was transferred onto two petri dish lids each and one of each (DMSO control, slow motor, and fast motor) was exposed to 365 nm light (UV-c) for 5 minutes (Fig. 2b and c). The distance between the light source and the bacterial culture was 1.3 cm (0.5 inches) (Fig. 2d). The other set was not exposed to light and was used as the non-activated control. After light activation, the bacterial cultures were serially diluted, plated on 60 × 15 mm Petri dishes with LB or MADC agar, and incubated overnight at 37 °C. Colonies were counted and the bactericidal effect of MNM on the different bacterial species was determined.

### 2.4. Bacterial viability assays with 395 nm light-activated MNMs

Using an overnight culture of wild-type *M. smegmatis* (mc^2^ 155 strain), a secondary culture was started in a 4 ml volume. OD_600_ of the overnight culture was measured. The starting OD_600_ of the secondary culture was 0.1. This culture was placed at 37 °C for about 3 h to give an active culture of *M. smegmatis*. Ending OD_600_ of the secondary culture was measured. A serial dilution of this culture was made using 100 μl of culture with 900 μl in MADC media. 1 ml dilutions of 10^−4^ cultures were used for the experiment. 1 μM concentration of MNMs in DMSO was used. Using a 10 mM working stock of MNMs, a 1 mM solution of MNMs was prepared with 2 μl of 10 mM MNMs with 18 μl of 1x PBS. 1 μl of this 1 mM MNMs was added to 1 ml of *M. smegmatis* culture at 10^−3^ to give a 1 μM concentration. 1 μl of DMSO without MNMs was added to the second 1 ml *M. smegmatis* culture at 10^−3^ as the control. The DMSO concentration for the experiment was 0.1 %. They were incubated for 30 minutes on a test tube rocker at room temperature. At 30 minutes after incubation, 100 μl of the culture of each was transferred into a 96-well plate with 4 replicates each. These were exposed to 395 nm light for 0, 5, 15, and 30 minutes. The light source was directly plated on top of the 96-well plate. After 395 nm light activation, serial dilutions of cultures were made and plated for CFU counts. These plates were incubated at 37 °C overnight for colony growth.

### 2.5. Confocal microscopy to observe MNM and M. smegmatis interactions

A confocal microscope (Nikon A1R+) with spectral capability, lasers FITC (fluorescein isothiocyanate) with an excitation wavelength of 488 nm and TRITC (tetramethylrhodamine isothiocyanate) excitation wavelength of 561 nm was used to detect excitation of BODIPY (505–512 nm) and tdTomato (575–585 nm), respectively. Using a 60x oil immersion objective, ψms23 (*M. smegmatis*:: tdTomato) were imaged 30 minutes post-exposure to MNM **3** and MNM **4** tagged with BODIPY. After exposure to MNM, ψms23 was washed with 1x PBS, fixed with 4 % paraformaldehyde, and washed again after fixation. 10 μl of No MNM, MNM **3,** and MNM **4** exposed ψms23 were then mounted on slides with 8 μl of mountant solution each. Each sample was imaged at eight different visual fields using a magnification of 600x and a resolution of 1024. Representative images are presented to show differences observed after exposure to non-activated and activated MNM.

### 2.6. MNM and M. smegmatis fluorescent intensity quantification

NIH ImageJ software was used to quantify the fluorescent intensities of images obtained by confocal imaging. Confocal images were taken with each laser (TRIC and FITC) individually and a merged image. Quantification was done on each of the wavelengths 488 nm and 561 nm and of the merged image. For each group, 8 images taken were quantified using the same area, and averages were calculated. For each group, the ratios of the MNM to *M. smegmatis* were obtained and their averages were calculated. The results presented are an average of 8 separate images taken for each group.

### 2.7. In vivo imaging system (IVIS) and quantification

IVIS Lumina II was used to quantify the total fluorescence of plates used for plating ψms23 post the day after carrying out viability assays. Each of the plates was imaged using an emission wavelength of 580 nm and an excitation wavelength of 535 nm. The IVIS settings were epi‑illumination, Bin 4 (medium), FOV: 12.5, f2 and exposure time of 0.5 s. Three replicates for each group were imaged. Living Image software was used to analyze and quantify the fluorescence intensity. A region of interest (ROI) was selected to include the whole plate and background fluorescence was reduced from each image to normalize readings from each plate. The units of measurement were radiance (photons) with units of p/sec/cm^2^/sr. All plates used for quantification are included in the image, with lower and upper limits of 4.5 × 10^7^ and 1.0 × 10^8^, respectively.

### 2.8. Statistical analyses

All experiments were carried out with at least an n of 3. The numbers of replicates used in each experiment are stated in each experiment if >3 were used. GraphPad Prism was used to perform two-tailed unpaired t-test statistical analyses to compare the means of different bacterial exposure groups. Means and standard error of the mean are presented in each of the graphs plotted in Microsoft Excel.

## 3. Results

### 3.1. Light-activated MNM 1 reduces the viability of M. smegmatis, M. tuberculosis, S. aureus, and E. coli

**Fig. 3.**
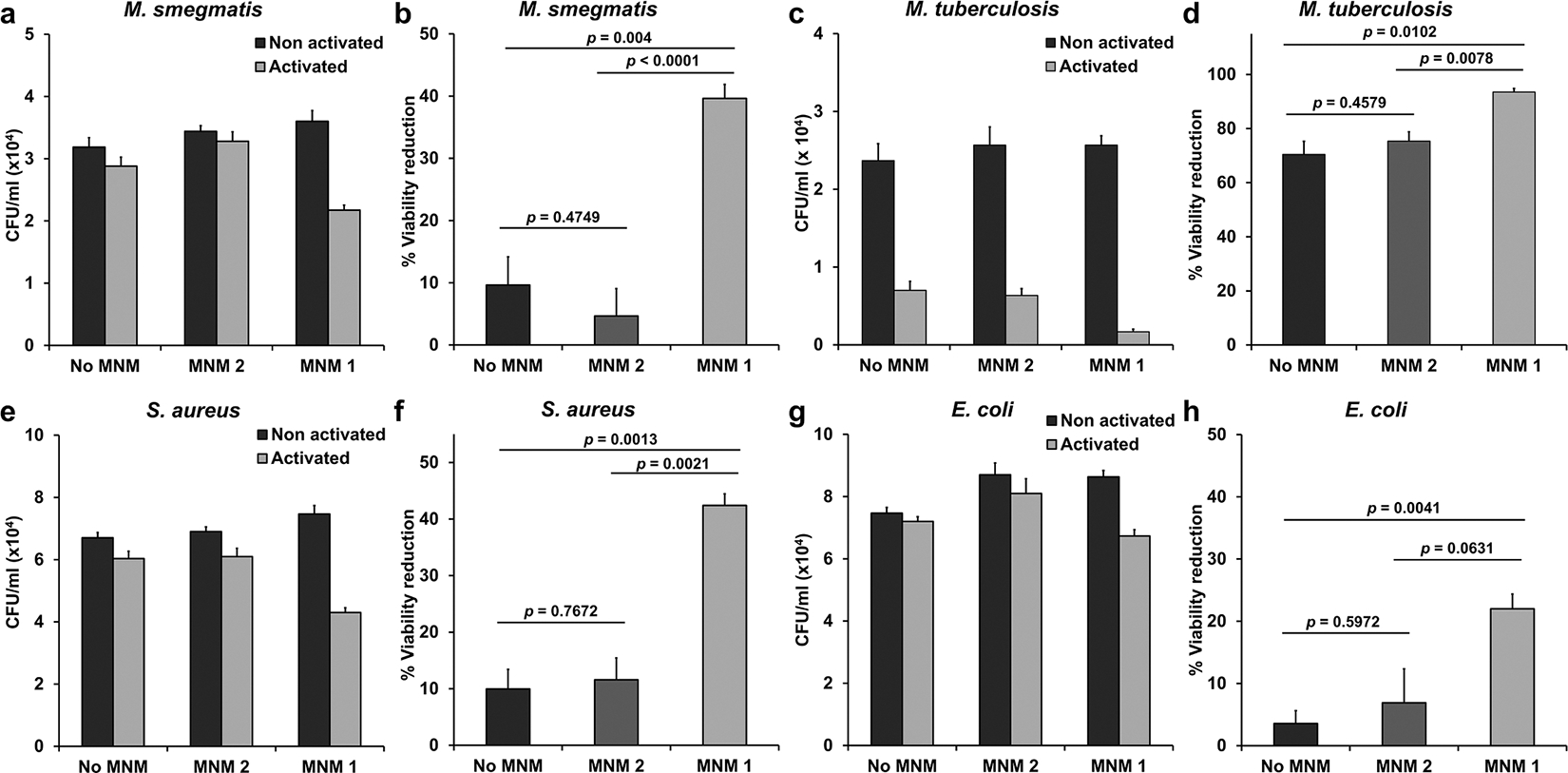
Viability reduction of bacteria with 365 nm light-activated molecular nanomachines (MNMs). Four different bacterial species were exposed to MNMs with 5 minutes of 365 nm light at 14 mW/cm^2^ (activated) and compared to the same samples not exposed to light (non-activated). No MNM is a 0.1 % DMSO control. MNM **2** is the slow motor. MNM **1** is the fast motor. (a) *M. smegmatis* (mc^2^ 155) and 1 μM of MNMs with or without 5 minutes of 365 nm light activation. (b) Light-activated No MNM, MNM **2** and MNM **1** groups show 10 %, 5 %, and 40 % reduction in viability of *M. smegmatis* compared to the non-activated controls, respectively. (c) *M. tuberculosis* (mc^2^ 7000) and 1 μM of MNMs with or without 5 minutes of 365 nm light activation. (d) Light-activated No MNM, MNM **2** and MNM **1** groups show 70 %, 75 %, and 93 % reduction in viability of *M. tuberculosis* compared to the non-activated controls, respectively. (e) *S. aureus* (Xen 36) and 1 μM of MNMs with or without 5 minutes of 365 nm light activation. (f) Light-activated No MNM, MNM **2** and MNM 1 groups show a 10 %, 12 %, and 42 % reduction in the viability of *S. aureus* compared to the non-activated controls, respectively. (g) *E. coli* (K-12) and 1 μM of MNMs with or without 5 minutes of 365 nm light activation. (h) Light-activated No MNM, MNM **2** and MNM **1** groups show 4 %, 7 %, and 22 % reduction in viability of *E. coli* compared to the non-activated controls, respectively. Error bars represent the standard errors of the mean.

We exposed *M. smegmatis* (mc^2^ 155) to 1 μM of MNM **1** with 5 minutes of 365 nm light-activation and observed a 40 % relative reduction in bacterial viability compared to a non-activated MNM **1** control (Fig. 3a and b). This reduction in bacterial viability is attributed to the action of activated MNM **1**, as 365 nm light alone, No MNM, MNM **2,** or non-activated MNM **1** showed little deleterious effect on *M. smegmatis* (≤ 10 % viability reduction). *M. smegmatis* was exposed to 1 μM of MNM **1**, as a higher concentration (1 μM) resulted in over 30 % viability reduction in *M. smegmatis* even without light (Supplemental Figure 2a). Similarly, we exposed *M. tuberculosis* (mc^2^7000) to 1 μM of MNM **1** with 5 minutes of 365 nm light-activation and observed a 93 % relative reduction in bacterial viability compared to a non-activated MNM **1** control (Fig. 3c and d). However, *M. tuberculosis* was much more sensitive to the deleterious effects of exposure to 5 minutes of 365 nm light, as both the No MNM and MNM **2** controls showed 70 % and 75 % viability reduction upon light activation. Despite the sensitivity of *M. tuberculosis* to 365 nm light, light-activated MNM **1** still significantly reduced bacterial viability compared to No MNM control (*p* = 0.0102) and MNM **2** (*p* = 0.0078). This shows that light-activated MNM **1** can significantly reduce the viability of both *M. smegmatis* and *M. tuberculosis*.

Mycobacteria are acid-fast bacteria and have a thick waxy layer on their cell wall that helps protect them from antibiotics and the host immune system [23]. We were interested in assaying the bactericidal properties of light-activated MNM **1** against Gram-positive and Gram-negative bacteria. Therefore, we exposed *S. aureus* (Gram-positive) and *E. coli* (Gram-negative) to MNM **1**. When *S. aureus* (Xen 36) was exposed to 1 μM of MNM **1** with 5 minutes of 365 nm light-activation and observed a 42 % relative reduction in bacterial viability compared to a non-activated MNM **1** control (Fig. 3e and f). No MNM, MNM **2** or non-activated MNM **1** showed little deleterious effect on *S. aureus* (≤ 12 % viability reduction). The reduction in viability was comparable to that observed in *M. smegmatis*. When *E. coli* was exposed to 10 μM of MNM **1** with 5 minutes of 365 nm light activation we observed a 22 % relative reduction in viability compared to a control without MNM **1** (Fig. 3g and h). No MNM, MNM **2,** or non-activated MNM **1** showed little deleterious effect on *E. coli* (≤ 7 % viability reduction). An MNM **1** concentration of 10 μM was used for *E. coli* as both non-activated 1 μM and 10 μM showed similar deleterious on *E. coli* (Supplemental Figure 2b). Compared to *M. smegmatis* or *S. aureus, E. coli* showed relatively less viability reduction with light-activated MNM **1** (22 % vs 40 %). These results show that a 5-minute light activation of MNM **1** significantly reduced viability of *M. smegmatis, M. tuberculosis, S. aureus* and *E. coli*, and have the potential to be used as a broad-spectrum antibacterial agent.

### 3.2. Light-activated MNM 5 reduces viability in M. smegmatis

**Fig. 4.**
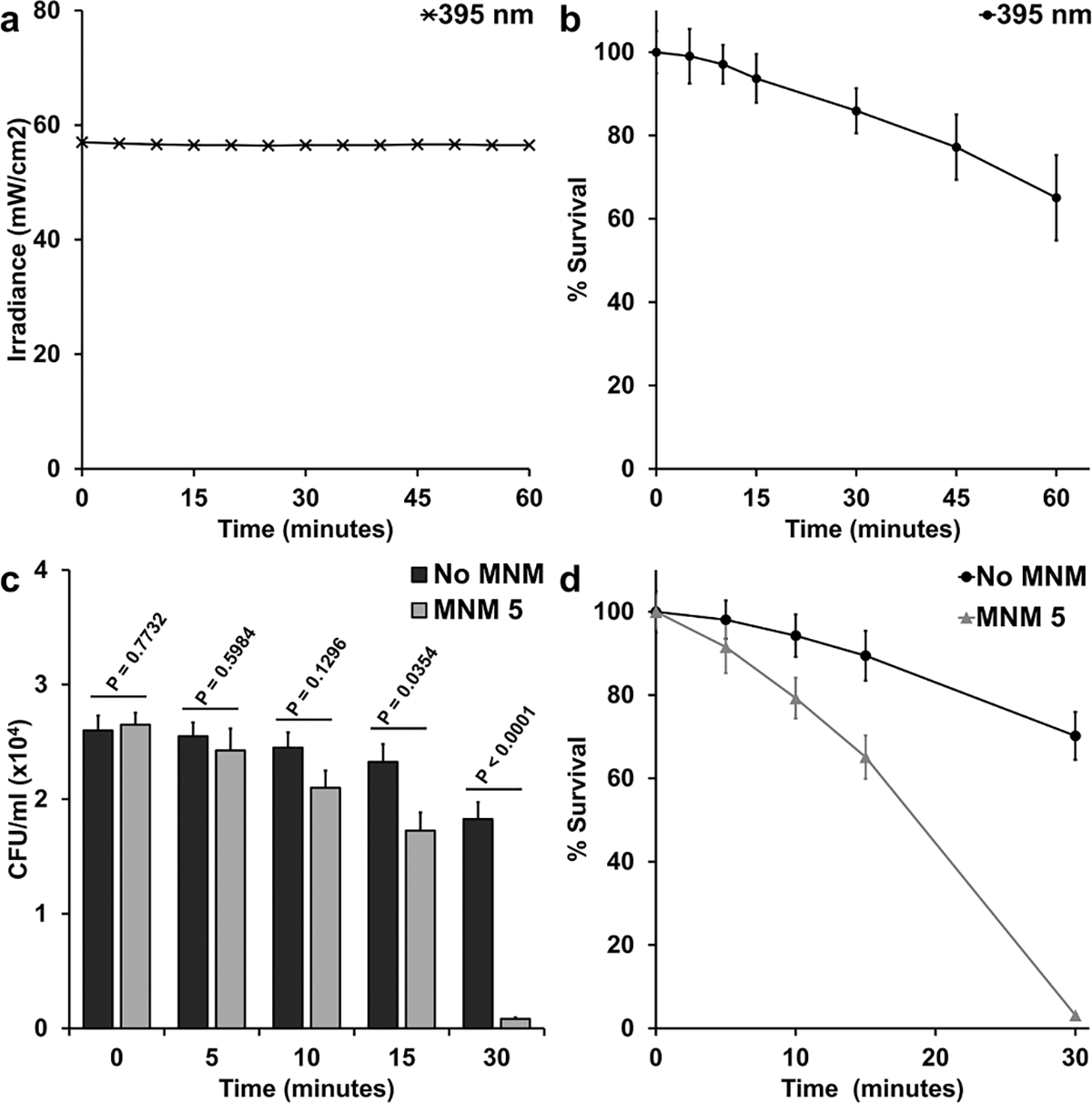
Viability reduction of *M. smegmatis* with 395 nm light-activated molecular nanomachines (MNMs). *M. smegmatis* (mc^2^ 155) was exposed to 1 μM of MNM **5** activated at different lengths of time to a 395 nm light source at 56.5 mW/cm^2^ to observe the reduction in bacterial viability caused by activated MNM **5**. (a) The irradiance of the 395 nm light source measured over one hour held steady within a range of 56.4 to 56.8 mW/cm^2^. (b) Bactericidal effects of the 395 nm light source at 56.5 mW/cm^2^ were observed over one hour. *M. smegmatis* percent viability compared to the starting culture at different lengths of light activation: 5 minutes (99 %), 10 minutes (97 %), 15 minutes (94 %), 30 minutes (86 %), 45 minutes (77 %), 60 minutes (65 %). (c) Viability reduction of *M. smegmatis* exposed to light-activated MNM **5** compared to a No MNM control exposed to the same duration of 395 nm light. At 15 and 30 minutes of light-activated MNM **5** showed a significant decrease in viability compared to the No MNM control. (d) Bactericidal effects of light-activated MNM **5**. *M. smegmatis* percent viability compared to the starting culture at different lengths of exposure to light-activated MNM **5**: 5 minutes (92 %), 10 minutes (79 %), 15 minutes (65 %), 30 minutes (3 %). Error bars represent the standard errors of the mean.

Light-activated MNM **1** reduced *M. smegmatis* viability by 40 % within 5 minutes. However, 365 nm light showed deleterious effects of *M. smegmatis* with prolonged exposure (Supplemental Figure 3a). Therefore, we were not able to reasonability assess the bactericidal potential of MNM **1** with prolonged exposure without reducing bacterial viability with the 365 nm light. To address this, we used a different fast motor, MNM **5**, that is activated with 395 nm light (Fig. 1d). Using a 395 nm light source that emitted 56.5 mW/cm^2^ of flux, we assess the deleterious effect of this source on *M. smegmatis* (Fig. 4a). Compared to the 365 nm light source, the 395 nm light had a smaller deleterious effect on *M. smegmatis* over one hour, reducing viability by 14 % at 30 minutes and 35 % at 60 minutes of light activation (Fig. 4b and Supplemental Figure 3). Therefore, we exposed *M. smegmatis* to 1 μM of MNM **5** and activated it for a period of 30 minutes, assessing the viability reduction of *M. smegmatis* at 5, 10, 15 and 30 minutes (Fig. 4c and d). *M. smegmatis* was also exposed to 395 nm light without MNM **5** (No MNM) as a control. *M. smegmatis* percent viability reduction due to light-activated MNM **5** at 5, 10, 15 and 30 minutes was 8 %, 21 %, 35 %, and 97 %, respectively. At 15 and 30 minutes of light-activated MNM **5** showed a significant decrease in viability compared to the No MNM control with *p*-value = 0.0354 and *p*-value < 0.0001, respectively. These results show that fast motor MNM **5** was able to reduce *M. smegmatis* viability by 97 % and indicate its potential as a clinically relevant antibiotic agent.

### 3.3. Light-activated fast motor MNM colocalizes with M. smegmatis

**Fig. 5.**
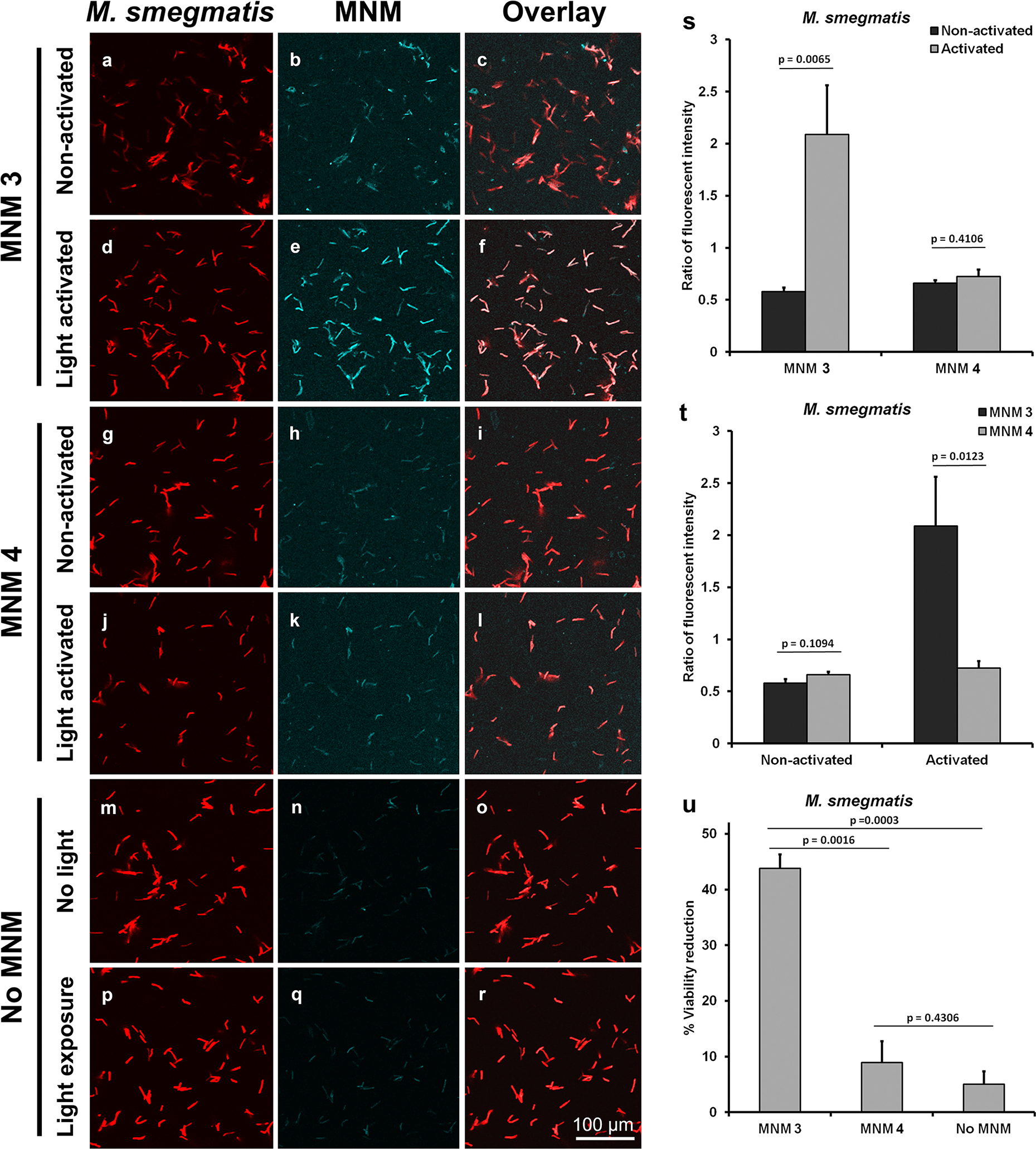
High-resolution confocal images show light-activated MNM **3** colocalize with *M. smegmatis. M. smegmatis* expressing tdTomato (ψms23) were exposed to 10 μM of fluorescent MNM-BODIPY and were imaged at 30 minutes after exposure to non-activated or light-activated MNM. Bacteria with MNM **3**, MNM **4,** or without MNM activated with 365 nm light for 5 minutes and compared with the non-activated group. A 60x oil-immersion objective was used with excitation wavelengths of 488 for BODIPY and 561 nm for tdTomato. (a-c) Non-activated MNM **3**. (d-f) Light-activated MNM **3**. (g-i) Non-activated MNM **4**. (j-l) Light-activated MNM **4**. (m-o) No MNM control without light activation. (p-r) No MNM control with light activation. Column *M. smegmatis* shows the bacterial imaged at 561 nm. Column MNM shows the BODIPY-MNM images at 488 nm. The overlay column shows the overlay of the *M. smegmatis* image and the MNM images for each group. (s-t) Confocal imaging quantification using ImageJ. (s) Comparison of MNM **3** and MNM **4** colocalized *M. smegmatis*. (t) Comparison of colocalization of MNM with and without light activation (u) tdTomato expressing *M. smegmatis* (ψms23) exposed to 10 μM of MNM. The relative reduction in viability of *M. smegmatis* exposed to light-activated MNM **3** and MNM **4**. Error bars represent the standard errors of the mean.

After characterizing the nanomechanical antimicrobial properties of light-activated MNM **1** and **5**, we were interested in studying the mechanism of action of these MNMs against *M. smegmatis*. For this, we used MNM **1** and MNM **2** with fluorophore BODIPY attached to them forming MNM **3** and MNM **4**, respectively (Fig. 1e and f). *M. smegmatis* strain expressing tdTomato fluorophore (ψms23) was exposed to light-activated MNM **3** and **4** and imaged with confocal microscopy (Fig. 5). We observed a 3x increase in fluorescent intensity in MNM-BODIPY levels in ψms23 exposed to light-activated MNM **3** compared to non-activated MNM **3** (*p*-value = 0.0065) (Fig. 5a-f and s). In contrast, there was no change in fluorescent intensity in MNM-BODIPY levels in ψms23 exposed to non-activated or light-activated MNM **4** (*p*-value = 0.4106) (Fig. 5g-l and s). Control ψms23 imaged without any MNM-BODIPY showed background fluorescent levels were minimal (Fig. 5m-r).

Overlay of ψms23 with MNM-BODIPY showed that light-activated MNM **3** colocalized or tightly associated with *M. smegmatis* compared to non-activated MNM **3** (Fig. 4b, c, e and f). This is seen by the change in color of *M. smegmatis* from red to a lighter color in the overlay image, representing colocalization (Fig. 5f). In contrast, colocalization was absent or occurred at low levels in MNM **4** and non-activated MNM **3** (Fig. 5c, i, l). Fluorescent intensity was also significantly higher in light-activated MNM **3** compared to MNM **4** (*p*-value = 0.0123) (Fig. 5t). The increase in colocalization indicates that light-activated MNM **3** is more tightly associated with *M. smegmatis,* either through embedding in the bacterial cell surface or entering the bacteria.

To confirm that MNM with attached fluorophore BODIPY (MNM **3**) had nanomechanical properties similar to MNM **1**, we carried out viability assays with conditions used for confocal microscopy. Light-activated MNM **3** showed a 35 % viability reduction when compared to a control without MNM, and a 30 % viability reduction compared to MNM **4** (*p*-value = 0.0003 and 0.0016, respectively) (Fig. 5u, Supplemental Figure 4). In addition to colony forming unit (CFU) counts, we also quantified the amount of fluorescent emitted by *M. smegmatis* colonies exposed to MNM **3**, MNM **4,** and no MNM control (Supplemental Figure 5). We observe a significant reduction in fluorescent signal in *M. smegmatis* exposed to light-activated MNM **3** compared to MNM **4** and no MNM control (*p*-value = 0.0013 and 0.0013, respectively) (Supplemental Figure 6). The observed reduction in fluorescent signals correlates with the reduction in bacterial viability (Fig. 5u). These results confirm that not only MNM **1**, but MNM **3** with large attachments (BODIPY fluorophore) on its stationary component still retains its nanomechanical antimicrobial properties. Our observations of confocal microscopy together with viability assays show that fast motor MNMs colocalize and tightly associate with *M. smegmatis* and cause a reduction in viability through its nanomechanical action on the bacteria. This imaging study provides a closer look at the mechanism of light-activated MNMs and adds to our understanding of how these MNMs carry out their nondrilling function on bacteria.

### 3.4. Model illustrating the nanomechanical antimicrobial action of MNMs

**Fig. 6.**
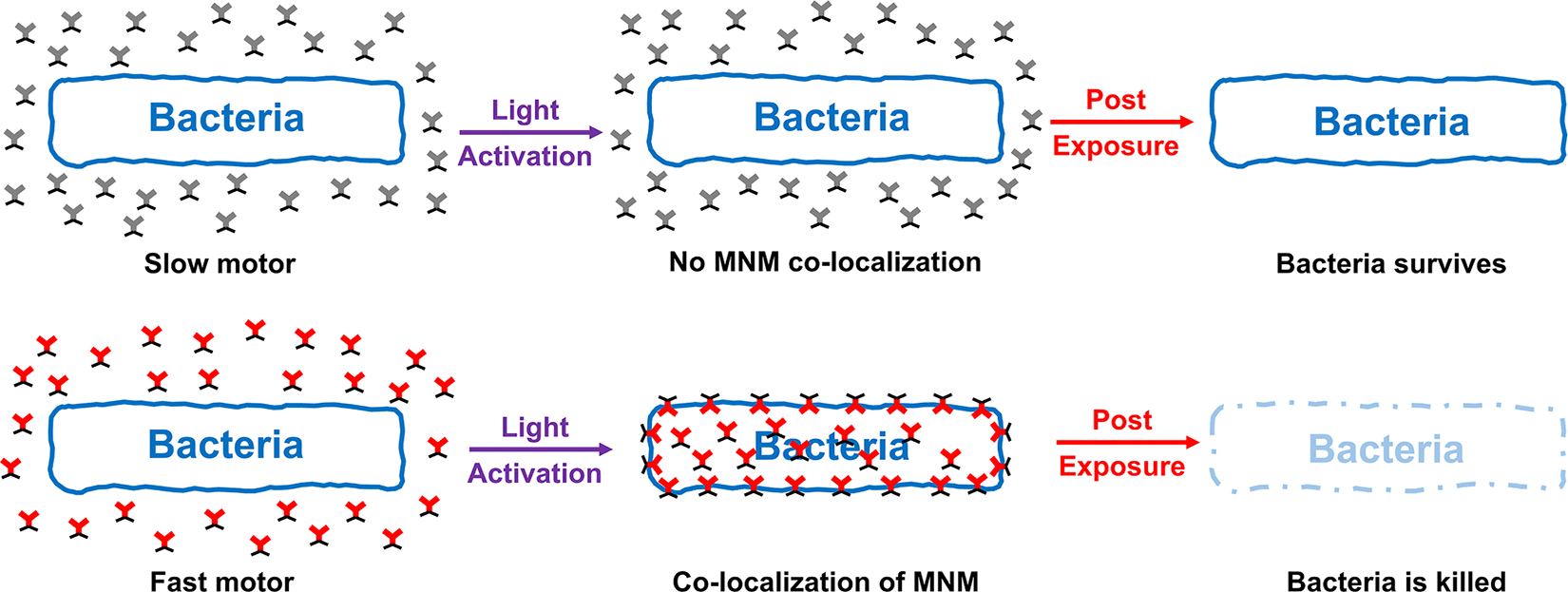
Model illustrating nanomechanical action of light-activated MNMs on bacteria. Bacteria exposed to MNMs have two distinct outcomes when activated by 365 nm light. Slow motors (MNM **2** and **4)** have no rotational action, remain outside the bacteria, and have little to no effect on bacterial viability. Whereas fast motors (MNM **1, 3,** and **5)** colocalize and embed in the bacterial cell wall causing disruptions that lead to a significant reduction in bacterial viability.

Our results show that fast motor MNM **1** and **3** when activated with 365 nm light, cause a reduction in bacterial viability in *M. smegmatis, M. tuberculosis, S. aureus,* and *E. coli*. This contrasts with the slow motor MNM **2** and **4** which did not show a significant reduction in bacterial viability with the same light activation. The confocal imaging study showed that MNM **3** colocalized with *M. smegmatis* with light-activation. Taking these observations together, we propose that upon light activation, the fast motor MNMs can embed and drill into bacterial cell walls through their nanomechanical action. This action causes disruptions in the bacteria cell walls resulting in the killing of the *M. smegmatis* bacterium. In contrast, slow motor MNMs are not able to penetrate the bacteria cell surface and do not have any bactericidal effects on the bacteria (Fig. 6).

## 4. Discussion

Antimicrobial resistance is a growing global crisis with significant public health and socio-economic implications [1,24]. The increasing prevalence of MDR and XDR pathogens necessitates the urgent development of alternative treatment strategies. Our research shifts antimicrobial treatment paradigms from biochemical interactions to a nanomechanical approach, leveraging the unique bactericidal properties of MNMs [13,14].

This study demonstrates that light-activated fast MNMs significantly reduce bacterial viability, achieving up to a 97 % reduction within 30 minutes. Unlike conventional antibiotics, it is unlikely for pathogens to develop resistance to a mechanical form of an antimicrobial agent, as the nanomachine did not need a specific target to act on. While antimicrobial resistance typically develops over extended periods, pathogenic bacteria did not develop resistance to MNMs in over 20 bacterial growth cycles [14].The MNMs used in this study also did not show cytotoxicity when exposed to J774A.1 macrophage cells up to a concentration of 100 μM [13].

Previous studies have demonstrated that MNMs disrupt bacterial cell walls through a nanomechanical drilling action, as confirmed by membrane permeability assays [13,14]. This study furthers our current understanding of the mechanism of action of light-activated nanomachines by showing that the fast-rotor MNMs colocalize with the bacteria upon activation, using fluorescently tagged MNMs (Fig. 6). MNMs are likely to first localize near the bacteria. When activated with light energy, they embed in the bacterial cell wall via their nanomechanical action, inducing structural damage and compromising bacterial membrane integrity. We showed this colocalization and internalization of MNM **3** with *M. smegmatis* upon light activation (Figs. 5 and 6) by fluorescence confocal microscopy.

The concentration of MNMs used for mycobacteria and *S. aureus* was 1 μM, while the concentration of MNMs used for *E. coli* was 10 μM. These concentrations were selected to optimize the action of MNM **1** while reducing their toxic effect on the bacteria (Supplemental Figure 2). *E. coli* was able to withstand a higher concentration of MNM. This could likely be due to the cell wall structure of Gram-negative bacteria compared to Gram-positive or acid-fast bacteria and is similar to what we have observed with *K. pneumoniae*, another Gram-negative bacteria [13].

Recent advances in the biosynthesis of metal nanoparticles, such as silver (AgNPs), gold (AuNPs), titanium dioxide (TiO_2_NPs), and zinc oxide (ZnONPs), have demonstrated potent antibacterial, antioxidant, and catalytic properties [25–27]. ZnONPs exhibit superior antibacterial potential over TiO_2_NPs, with the added advantage of negligible cytotoxicity, reinforcing their suitability as antimicrobial agents [28]. Additionally, AgNPs synthesized using green chemistry approaches have demonstrated significant catalytic activity, particularly in environmental remediation [29]. The increasing concerns of microplastic pollution and its impact on microbial communities further underscore the necessity of nanoparticle-based antibacterial strategies [30].

The incorporation of MNMs as antibacterial agents aligns with these nanoparticle-based advancements, as they provide mechanical bactericidal effects independent of biochemical interactions. Our findings highlight that light-activated MNMs cause substantial bacterial membrane disruption, a mechanism distinct from conventional antibiotics and to the action of ROS-generating nanomaterials [31]. By integrating nanomechanical antimicrobial strategies with existing nanotechnology-based approaches, MNMs offer a novel and effective pathway to combating MDR and XDR bacterial infections.

There are some limitations of this study. First, we use 365 nm light which is in the range of UV-c and it can have some harmful effects on living tissue. This limited the exposure time we could use to activate the MNM. Of the bacteria used in this study, *M. tuberculosis* was especially sensitive to 365 nm light activation over 5 minutes. We predict that with the increase of MNM activation time, the antimicrobial effect of MNM may increase several-fold as we saw with the 395 nm activated MNM **5**. The sensitivity to 365 nm light is expected because *M. tuberculosis* is not an environmental mycobacteria and does not produce the pigments that protect environmental mycobacteria from UV (sun) light [32]. Therefore, UV light is used in clinical or environmental settings to control to spread of *M. tuberculosis* and other pathogenic mycobacteria that do not provide protective pigments [33–35]. This explains our observations that *M. tuberculosis* was much more sensitive to 365 nm light compared to *M. smegmatis*.

Second, 365 nm light has relatively low penetration in host organs and tissue. This limits the effectiveness of MNM for the treatment of deep tissue infections. However, with our current approach, MNM can be potentially used as an antimicrobial agent to treat local infections including skin, mouth, lung, and urinary tract infections. In each of these target organs, MNM can be activated with light from an external source. However, to address both the above concerns, our group is currently working on the next generation of MNM that can be activated by visible light and near-infra-red (NIR) light. This will greatly increase the ability of MNM activation in much deeper host targets and also allow the activation of MNM for a longer time to achieve a far superior antimicrobial efficacy, without any harmful effects on the host.

Third, in this study, we used MNM which was only the motor component, without any specific binding affinity to the bacteria. The lack of specificity towards target bacteria may also explain the relatively low reduction in viability that we observed. When MNM **1** was used to target and permeabilize cancer cells, they had short-sequence peptides that allowed selective binding and high specificity [36]. Targeting specific bacterial receptors can be achieved by selecting specific ligands on the complex cell wall structure in bacteria [37]. Studies have shown that globotriaosylceramide (GB3) and lactosylceramide ligands bind LecA receptors with specificity in *Pseudomonas aeruginosa* and can be used for drug delivery in these pathogens [38,39]. We propose that by combining MNMs with pathogen-specific ligands, their specificity and attachment efficiency to target bacteria could be enhanced, improving antimicrobial efficacy. Increasing MNM attachment to bacteria enhances nanomechanical damage, thereby improving its efficacy as an antimicrobial agent.

This study provides evidence that light-activated fast MNMs effectively reduce bacterial viability, particularly in mycobacteria, positioning them as a promising approach to combat antibiotic resistance. The demonstrated efficacy of MNMs against *M. smegmatis, M. tuberculosis, S. aureus*, and *E. coli* underscores their broad-spectrum potential. These findings hold clinical implications for addressing the growing challenges of antibiotic resistance in mycobacterial infections. In particular, the promise of MNMs for the treatment of MDR, XDR, and TDR *M. tuberculosis* strains is supported by our observations. While challenges remain in clinical translation, ongoing advancements in long-wavelength-activated MNMs present a viable path toward future antimicrobial interventions [40]. The unique nanomechanical action of MNMs offers an effective and potentially disruptive solution in the battle against multidrug-resistant pathogens.

## Supplementary Material

Supplementary Material

## Abbreviations:

AMR: Antimicrobial-Resistant
MDR: Multidrug-Resistant
XDR: Extensively Drug-Resistant
TDR: Totally Drug-Resistant
ESKAPE: *pathogens, Enterococcus faecium, Staphylococcus aureus, Klebsiella pneumoniae, Acinetobacter baumannii, Pseudomonas aeruginosa*, and Enterobacter species
MNMs: Molecular Nanomachines
EM: Electron Microscopy
RNA-seq: RNA Sequencing
M. smegmatis: Mycobacterium smegmatis
M. tuberculosis: Mycobacterium tuberculosis
M. leprae: Mycobacterium leprae
E. coli: Escherichia coli
ADC: Albumin Dextrose Catalase
LB: Luria-Bertani
DMSO: Dimethyl Sulfoxide
PBS: Phosphate Buffered Saline
CFU: Colony Forming Units
FITC: Fluorescein Isothiocyanate
TRITC: Tetramethylrhodamine Isothiocyanate
IVIS: In Vivo Imaging System
ROI: Region of Interest
NIR: Near-Infrared
GB3: Globotriaosylceramide
LecA: *Pseudomonas aeruginosa* Lectin A
