# Supplementary material for: Microbicidal mechanisms for light-activated molecular nanomachines in Mycobacterium smegmatis: A model for pathogenic bacteria
Source: OpenNano. Author manuscript; Available in PMC 2026 Feb 25. (PMC12392803; doi:10.1016/j.onano.2025.100240)
Supplement: Supplementary Material [file NIHMS2074297-supplement-Supplementary_Material.pdf]

Microbicidal Mechanisms for Light-Activated Molecular Nanomachines in *Mycobacterium smegmatis*: A Model for Pathogenic Bacteria

SUPPLEMENTAL MATERIAL

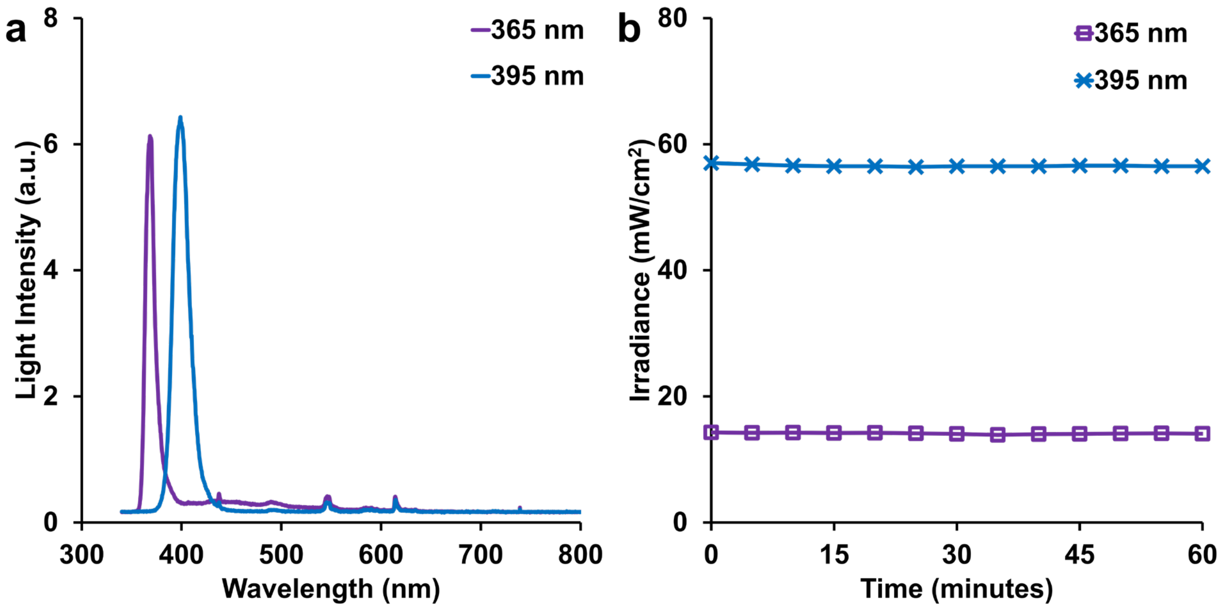

Supplemental Figure 1. The emission spectra of the 365 nm and 395 nm light sources. A spectrum test was performed on the 365 nm and 395 light sources used in this study, using a spectrograph. The 365 nm light source had an emission spectrum from about 358 to 386 nm, with a peak intensity at 368 nm wavelength. The 395 nm light source had an emission spectrum from about 382 to 422 nm, with a peak intensity at 398 nm wavelength.

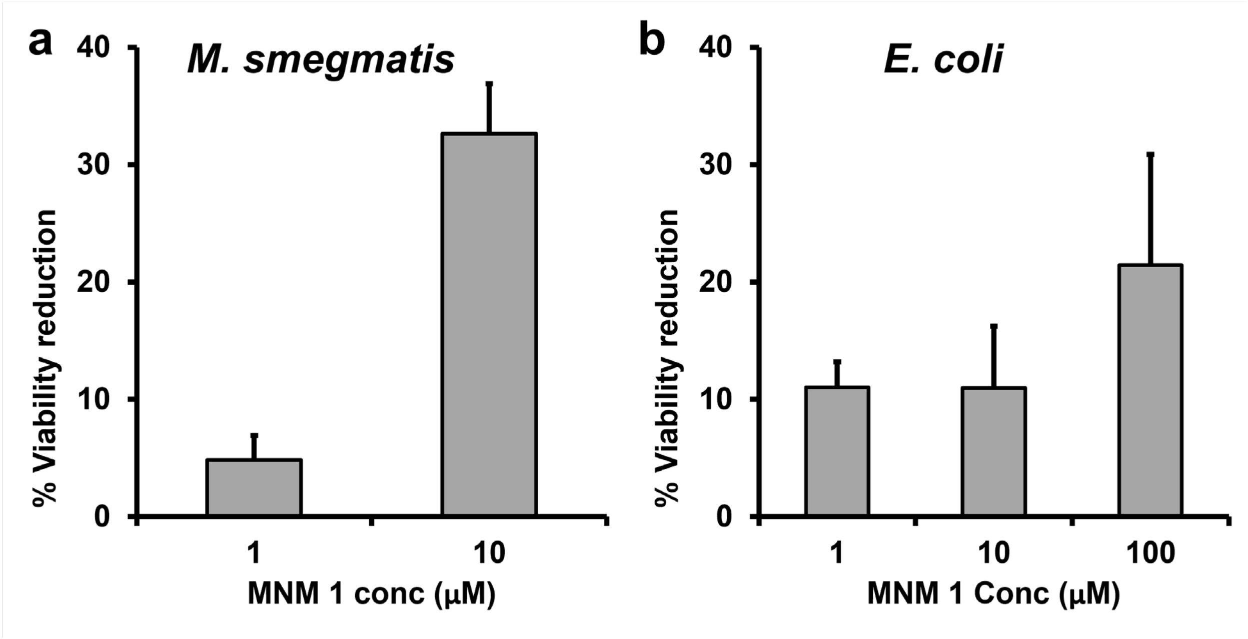

Supplemental Figure 2. MNM1 exposed to *M. smegmatis* and E. coli at different concentrations. (a) *M. smegmatis* exposed to 1 µM and 10 µM of non-activated MNM **1** displayed 5% and 33% of viability reduction, respectively. (b) *E. coli* exposed to 1 µM, 10 µM and 100 µM of non-activated MNM **1** displayed 11%, 11%, and 21% of viability reduction, respectively.

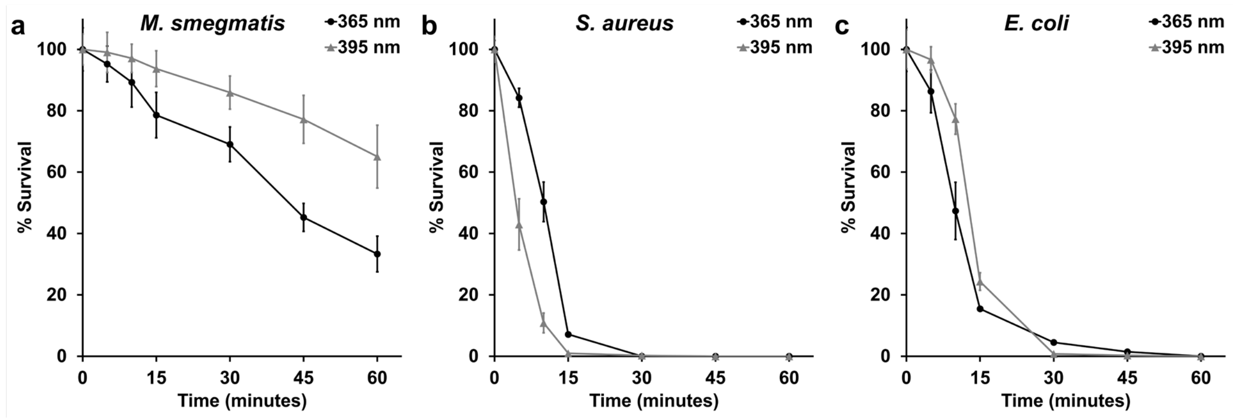

Supplemental Figure 3. Bactericidal effect of the 365 nm and 395 nm light sources on *M. smegmatis*, *S. aureus,* and *E. coli* over 60 minutes of constant light exposure.

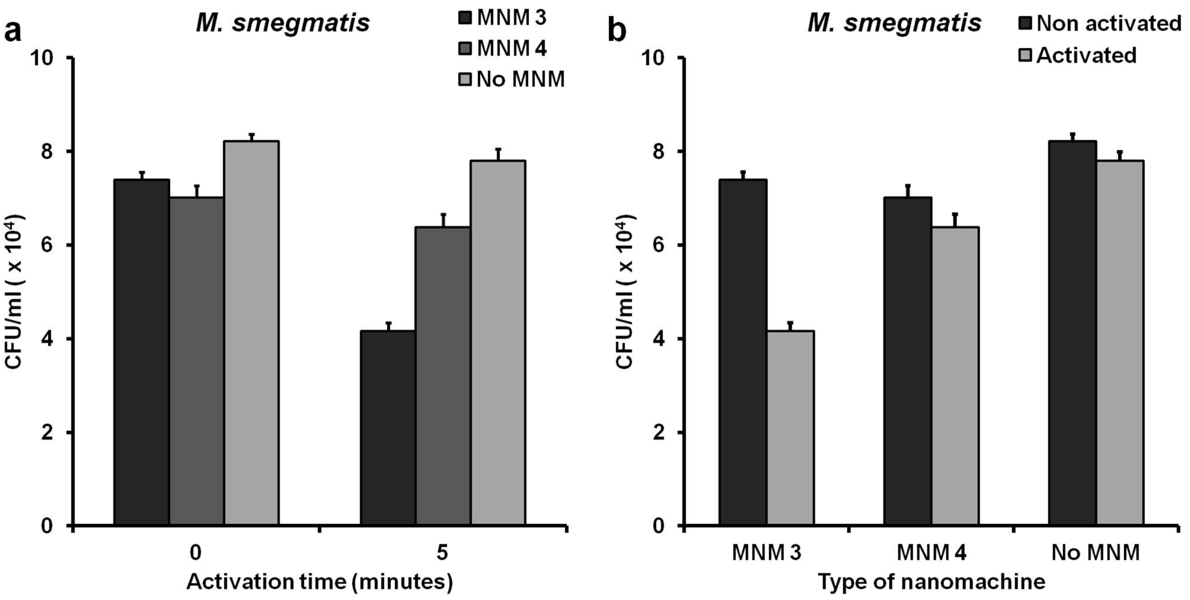

Supplemental Figure 4. Viability in CFU/ml of *M. smegmatis* (ψms23) in Activated to Non-Activated MNM

tdTomato expressing *M. smegmatis* (ψms23) exposed to 10 μM of MNM. (a) Comparison of ψms23 CFU/ml in MNM **1**, MNM **2,** and No MNM groups. (b) Comparison of ψms23 CFU/ml with and without light activation.

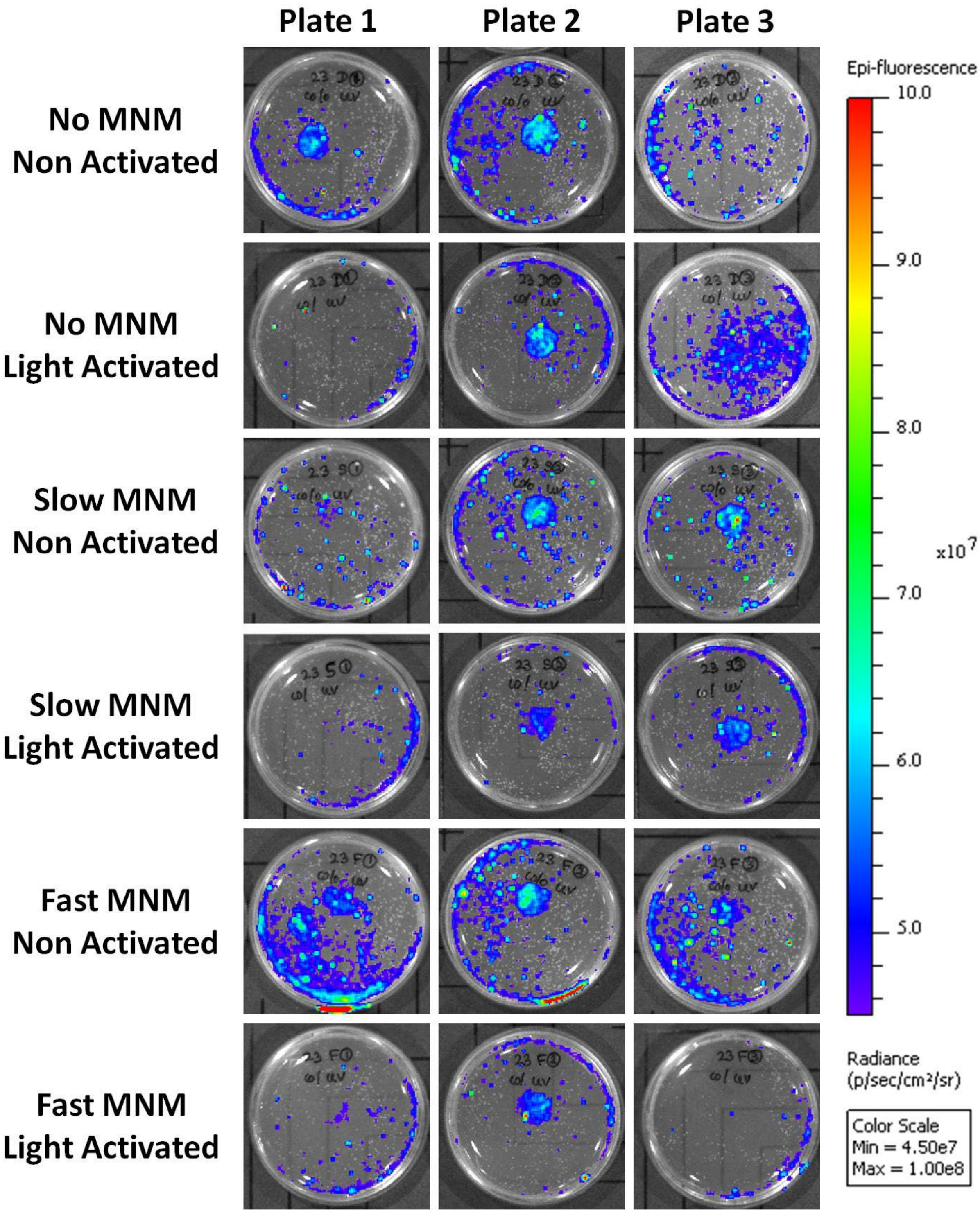

Supplemental Figure 5. IVIS Image of tdTomato Fluorescent

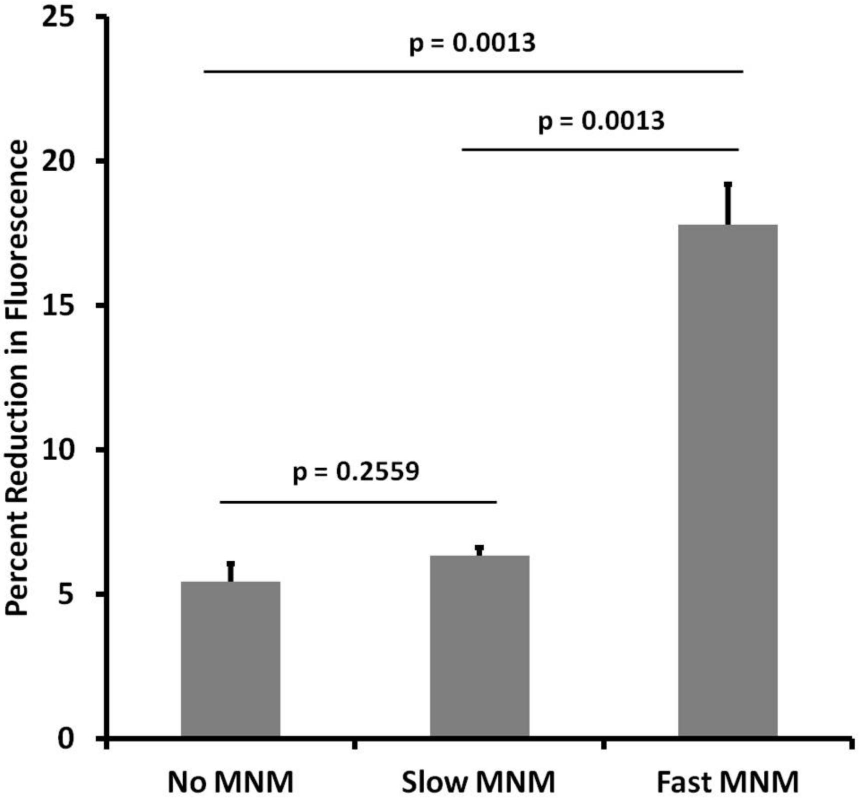

Supplemental Figure 6. Relative Reduction in *M. smegmatis* (ψms23) in Activated to Non-Activated MNM
